# The Role of Surfaces in Gas Transport Through Polymer Membranes

**DOI:** 10.3390/polym11050910

**Published:** 2019-05-20

**Authors:** Giuseppe Firpo, Elena Angeli, Patrizia Guida, Denise Pezzuoli, Diego Repetto, Luca Repetto, Ugo Valbusa

**Affiliations:** Nanomed Labs, Physics Department, University of Genova, Via Dodecaneso, 33, 16146 Genova, Italy; elena.angeli@unige.it (E.A.); patrizia.guida@unige.it (P.G.); denise.pezzuoli@edu.unige.it (D.P.); diegorepet@gmail.com (D.R.); luca.repetto@unige.it (L.R.); valbusa@fisica.unige.it (U.V.)

**Keywords:** adsorption rate, gas permeation, PDMS, diffusivity, solubility

## Abstract

This paper describes a procedure to measure the permeability *P*, diffusivity *D,* and rate of adsorption *k_1_*, thus determining the solubility *S* and rate of desorption *k_2_* of He, N_2_, O_2_, CH_4_, and CO_2_ on a polydimethylsiloxane (PDMS) membrane. The described procedure is able to determine experimentally all the physical quantities that characterize the gas transport process through a thin rubber polymer membrane. The experiments were carried out at room temperature and at a transmembrane pressure of 1 atm. The results are in good agreement with the available data in the literature and offer an evaluation of *k_1_* and *k_2_*.

## 1. Introduction

Membrane technology is a promising green technology that can solve several problems such as waste water treatment or desalination, CO_2_ capture in fuel power plants, gas pure production technology, gas sensing, etc. The complete understanding of gas transport through polymer films is still a challenge. Different models, depending on the gas/membrane system, have been developed to describe this phenomenon: Knudsen or Poiseuille diffusion for porous membranes, molecular sieve effects, solution–diffusion (SD) for dense membranes, with dual-mode sorption theory in case of glassy polymers [1]. The experimental results confirm the models in the range of validity of the theory. Considering the permeation of a gas through a dense polymer, the SD model [2,3] describes quite well this phenomenon and is routinely employed to determine the parameters that characterize a membrane, such as permeability *P*, diffusivity *D,* and solubility *S*. However, the behaviour of polymeric materials with respect to gas permeation depends on a great number of variables, including method of fabrication, composition of the polymeric matrix, glass transition temperature, grade of confinement. Consequently, it can happen that the simple SD model does not describe accurately the experimental results. In fact, works that denote some anomalies in the measurements of these quantities are not rare in the literature. This occurs when the permeability of a polymer to gases is measured by using a constant-volume variable-pressure (manometric) method and a constant-pressure variable-volume (volumetric) method, as Lundstrom reports in a recent review [4]. Several of these discrepancies could be avoided by following the procedure suggested by the American Society for Testing and Materials (ASTM International) in one of their reports on standard test methods to determine gas permeability of plastic films and sheeting [5]. This designation highlights that *P* should not be used unless its constancy with different membrane thicknesses has been verified, implying the possibility that permeability may be thickness-dependent. However, also following all recommendations of the previous designation to determine the values of the physical quantities that describe the mechanism of gas transfer along the membrane, remain some discrepancies between experimental and theoretical results.

In this paper, we focused our attention on the role of surface in the gas transport behaviour in dense polymer membranes. The paper investigated polydimethylsiloxane (PDMS), which is the most permeable rubbery polymer and can also be used as a rubber polymer model system [6]. Several authors [6,7,8,9,10,11] have already pointed out that, for these kinds of membranes, the overall mechanism of gas transport can be fully characterized only if the SD model is generalized by assuming non-equilibrium conditions at the upstream and downstream surfaces and by introducing the adsorption and desorption coefficients *k_1_* and *k_2_* to describe the dynamics of the adsorption process (SD*k_1_k_2_* model). In the present experiments, we used a dynamic apparatus that measures the gas transmission rate through a membrane of PDMS subjected to a known differential partial pressure of the tracer gas (He, N_2_, O_2_, CH_4_, and CO_2_). By following the electrical analogy proposed in reference [12] and by using the experimental set up of reference [6], this paper determined permeability *P* and rate of adsorption *k_1_*. In addition, a lag-time method measured diffusivity *D*. The knowledge of *D* permitted the calculation of solubility *S* and of desorption rate *k_2,_* allowing to determine all the physical quantities that characterize the permeation process. The paper reports the surface kinetic parameters, *k_1_* and *k_2_*, as a function of Lennard–Jones temperatures for all gas tested. The dependence of solubility and solubility-selectivity on gas critical parameters is also reported and compared with theoretical considerations [13].

### 1.1. Gas Transport Model

**Figure 1:**
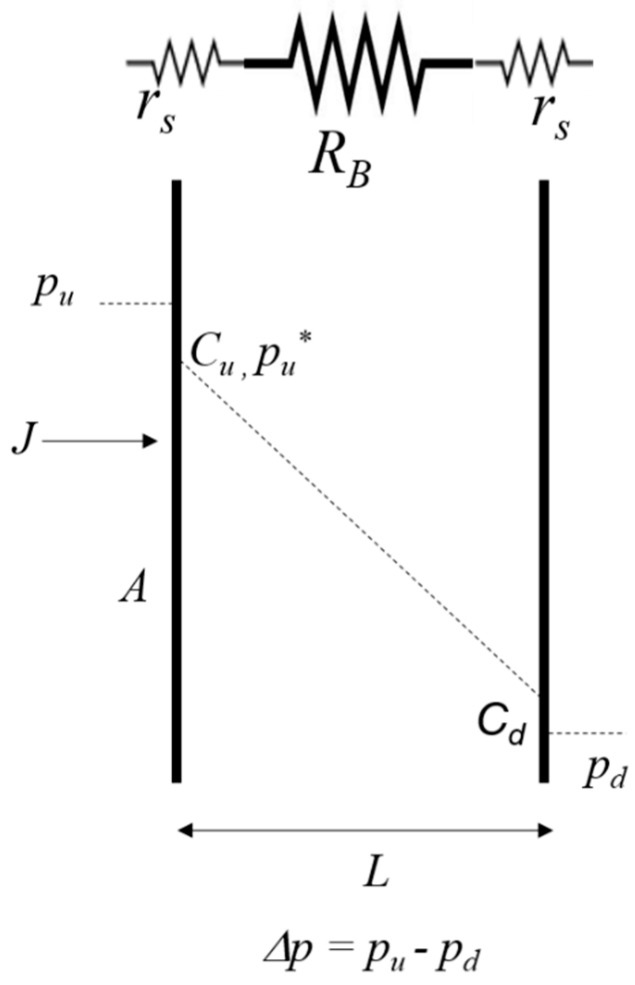
Electric analogy of the permeation process.

The SD*k_1_k_2_* model considers the permeation of a gas through a dense polymer membrane as a three-step mechanism: (1) sorption of the gas on the upstream surface of the membrane, (2) diffusion through the bulk of the membrane driven by a gradient concentration, (3) desorption through the downstream surface of the membrane. Considering the first step, the molar flux *J* in mol m^−2^ s^−1^ at the interface is given by:

(1) $$J=k_{1}p_{u}-\;k_{2}C_{u}$$

where *p_u_* is the upstream pressure, and *C_u_* the upstream concentration in the membrane at the first interface (see Figure 1); *k_1_* is expressed in mol m^−2^ s^−1^ Pa^−1^, *k_2_* in m s^−1^, and *C_u_* in mol m^−3^. Under the hypothesis of Henry’s law, *C_u_* can be expressed as *S* × p_u_*, where *S* = *k_1_*/*k_2_* is the solubility, and p_u_* is the upstream pressure in conditions of equilibrium at the interface. Consequently, Equation (1) can be written as:

(2) $$J=k_{1}p_{u}-\;k_{1}p_{u}{}^{*}=\;k_{1}\Delta p_{u}$$

where Δ*p_u_* = (p_u_ – p_u_^*^). From equation (2), it is possible to write Ohm’s law (by unit of membrane area *A*) for the case of a gas surface sorption on the polymer membrane:

(3) $$\frac{\Delta p_{u}}{J}\;=\;\frac{1}{k_{1}}=r_{s}$$

where *r_s_* is the surface resistance. Recent papers report a complete analysis of the phenomenon considering non-equilibrium at the surfaces [4,6,8]

With the same previous electric analogy applied to steps (2) and (3), the overall permeation process can be represented by the following equation that corresponds to the Ohm law’s in the case of three resistances in series (Figure 1):

(4) $$\frac{\Delta p}{J}\;=\;\frac{1}{k_{1}}\;+\;\frac{1}{P}\;+\;\frac{1}{k_{1}}$$

In this case, Δ*p* = (*p_u_ – p_d_*), *L/P* is the bulk resistance *R_B_*, where *P* is the product of diffusivity *D* by solubility *S* (Fick’s law). For *L*→0, *R_B_*→0, and the permeation process is limited only by the two upstream and downstream surface resistances *r_s_*. On the contrary, for great thicknesses, *r_s_* is negligible with respect to *R_B_*, and Equation (4) becomes

(5) $$\frac{\Delta p}{J}\;=\;R_{B}$$

Following reference [6], a thickness above 100 µm warranties that the surface resistance will be negligible.

A membrane of area *A* and thickness *L* exposed to a transmembrane pressure Δ*p*, experiences a flow *J*; *p_u_* and *p_d_* are, respectively, upstream and downstream pressure, and p_u_* is the upstream pressure in conditions of equilibrium at the interface. *C_u_* and *C_d_* are the upstream and downstream concentrations in the membrane at the interface. The dotted line in Figure 1 represents the linear variation of the concentration in the bulk (diffusivity is supposed to be independent from concentration). *R_B_* and *r_s_* are bulk and surface resistances, respectively, defined as in Equations (5) and (3).

### 1.2. Experimental Method

The present experiments measured the molar flux *J* across the membrane at room temperature, with Δ*p* =1 atm. The procedure used to determine *P* and *k_1_* is reported below. We first measured *P* for a membrane with *L* >> 100 µm. In this case, by measuring the quantity *ϕ* = Δ*p*/*J*, one obtains:

(6) $$P\;=\;\frac{L}{\phi }$$

Second, we measured the same quantity for a membrane with thickness *L* << 100 µm. In this case, Equation (4) holds, and *k_1_* is easily determined:

(7) $$k_{1}=\frac{2P}{\phi P-L}$$

By using the previously measured value of *P*, *k_1_* can be determined by a measurement of *ϕ.*

By measuring *ϕ* for two membranes with different thicknesses, it is therefore possible to measure *P* and *k_1._*

Diffusivity can be obtained by the lag-time method. Supposing to admit a gas at the upstream side of a membrane at *t* = 0, the equations of diffusion [14] give the following expression for the diffusivity:

(8) $$D=\frac{L^{2}J_{ss}}{6\left(J_{ss}t^{*}-\;\overset{t*}{\underset{0}{\int }}J\left(t\right)dt\;\right)}$$

where *J_SS_* is the gas flow at steady state, and t^*^ is greater than or equal to the time to reach the steady state. Considering that *S = k_1_/k_2_ = P*/*D:*

(9) $$k_{2\;}=\;k_{1}\frac{D}{P}$$

## 2. Materials and Methods

PDMS was the rubbery polymer used to measure surface kinetics parameters, and the tracer gases were: CO_2_, N_2_, He, O_2_, and CH_4_. The experimental setup employed to measure *ϕ* and diffusivity was the same reported in references [6,12], as was the assembling of the sample that guaranteed the same exposed area on the upstream and downstream sides. The apparatus was a high-vacuum chamber, where *J* and Δ*p* were measured by means of pressure gauges with high accuracy. The method measured transient fluxes *J* directly as the product of the pressure of the vacuum chamber by the pumping speed of the system to the tracer gas. The tracer gases were tested without a systematic order because none of them created irreversible effects on PDMS (CO_2_ plasticization occurs only for glassy polymers, for example). However, we waited for a complete degassing of the polymer between two measurements with different tracer gases. The membranes tested had two different thicknesses, i.e., 2 mm and 10 µm, to measure *P* in the first case and *k_1_* from Equation (7).

**Figure 2:**
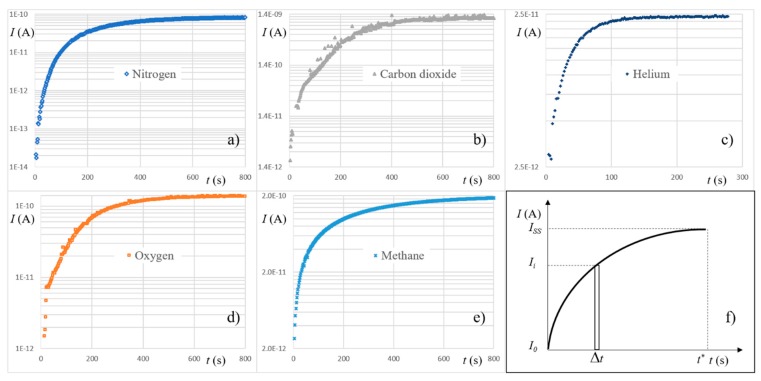
Diffusivity measurements. (**a**–**e**) Ion currents vs time of the tracer gases after filling the upstream chamber a *t* = 0 (*p_u_* = 10^5^ Pa); (**f**) representation of the parameters of Equation (10); in our case Δ*t* = 2 s.

By means of a residual gas analyzer (RGA) (purchased from Stanford Research Systems 1290-D Reamwood Ave. Sunnyvale, CA, USA), we measured diffusivity. Considering that the value of *J* depends on the value of the ion current *I* of the RGA and changing the integral with a finite sum of resolution equal to the sampling time of RGA (see Figure 2), Equation (8) could be rewritten in the following form:

(10) $$D\;=\;\frac{L^{2}\left(I_{ss}-\;I_{0}\right)}{6\left[\left(I_{ss}-\;I_{0}\right)t^{*}-\;\Delta t\sum_{i=1}^{n}\left(I_{i}-\;I_{0}\right)\right]}\;$$

where *I_SS_* and *I_0_* are the ion currents of the tracer gas at steady state and at the background, respectively, Δ*t* is the RGA sampling time, and *n* is the number of samples of ion currents equal to t*/Δ*t*. Measurements of permeability and diffusivity were always taken for pure gases (purity grade N5.0, minimum purity 99.999%).

The method to measure *ϕ* was the one reported in reference [11]; in this case the error for *L* was negligible with respect to that for gas flow *J.* For this reason, the percentage errors for *ϕ* and *P* were always less than 10%. The accuracy of *D* depended only on the accuracy of the ion current (see Equation (10)) and was always less than 10%. 

## 3. Results and Discussion

Table 1 reports the values of *P* and *k_1_* measured following the previously described procedure. The values of *P* were in good agreement with those reported in the literature [6,15,16].

The lag-time method allowed the determination of diffusivity of all five gases by means of RGA measurements (*L* = 2 mm). Figure 2 reports the behavior of the ion currents of the tracer gases from the filling of the upstream chamber (*t* = 0) up to steady state.

The measured values of *D* are reported in Table 1. *S* and *k_2_* were obtained by using Equations (8) and (9).

**Figure 3:**
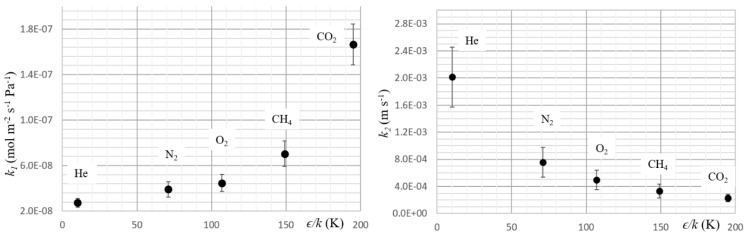
Surface kinetics parameters vs Lennard–Jones temperature. Graphs show the dependence of *k_1_* and *k_2_* on Lennard–Jones temperature *ϵ*/*k*.

The fundamental process for gas dissolution in a polymer is the condensation of the gas on the polymer surface. Gas solubility is determined by measuring the penetrant gas condensability through Lennard–Jones temperature *ϵ*/*k,* where *ϵ* is the depth of the potential well of the Lennard–Jones potential, and *k* is the Boltzmann’s constant. Because solubility is the ratio of *k_1_* and *k_2_*, it is interesting to report this parameter as a function on *ϵ*/*k.* Figure 3 shows this relationship.

**Figure 4:**
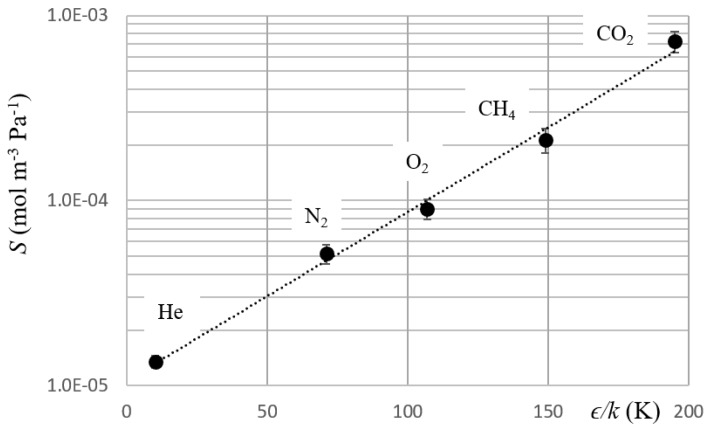
Solubility vs *ϵ/k*. The graph reports solubility as a function of Lennard–Jones temperatures.

The graphs in Figure 3 exhibit the dependence of sorption and desorption rates on Lennard–Jones temperatures. A high value of Lennard–Jones temperature facilitates the binding of gas molecules with the membrane surface and, consequently increases gas condensability and solubility. This behavior is intuitively the opposite for the desorption rate, as demonstrated by the graph for *k_2_* in Figure 3. The results were in accord with the thermodynamic approach developed by Teplyakov et al. [17] which gives the following expression for the solution of a gas in polymers:

Ln *S* = *N ×ϵ/k* + constant (11)

where *S* is the solubility, and *N* is a coefficient. Freeman [13] pointed out that *N* has the same value for many materials and indicated 0.023 as its value for a variety of liquids, rubbery polymers, and glassy polymers. In Figure 4, we reported *S* as function of *ϵ*/*k*.

The best-fit analysis of the curve of Figure 4 gave N = 0.021 ± 0.001 and constant = −11.4 ± 0.1 with an Adjusted R-Square = 0.991 (Adjusted R-Square is a modified version of the coefficient of determination that qualifies the linear regression; it also considers the number of predictors in the fitted line). These values were in good agreement with those reported in the literature [18,19].

## 4. Conclusions

This paper describes a procedure to evaluate all the characteristic parameters of the gas permeation process, i.e., *P*, *D*, *k_1_, k_2_*. The method was carried out for simple molecules, such as He, N_2_, O_2_, CH_4_, and CO_2_ permeating a PDMS membrane. The results are in excellent agreement with those reported in the literature. The values of *k_1_* and *k_2_* depend on Lennard–Jones temperatures of the studied gases, showing that a high value of Lennard–Jones temperature facilitates the binding of gas molecules with the membrane surface and, consequently increases gas condensability and, finally, solubility.

## Figures and Tables

**Table 1:** Permeability *P*, sorption rate *k_1_*, diffusivity *D*, solubility *S*, desorption rate *k_2_*, Lennard–Jones temperature *ϵ*/*k.* The transmembrane pressure was Δ*p* = 1.013 × 10^5^ Pa. The temperature was *T* = 293 K; *data were taken from reference [13].

Gas	*P* × 10^13^ (mol m^−1^ s^−1^ Pa^−1^)	*k_1_* × 10^8^ (mol m^−2^ s^−1^ Pa^−1^)	*D* × 10^9^ (m^2^ s^−1^)	*S* × 10^4^ (mol m^−3^ Pa)	*k_2_* × 10^4^ (m s^−1^)	*ϵ*/k* (K)
**He**	2.3 ± 0.1	2.7 ± 0.4	16.8 ± 0.3	0.14 ± 0.01	20 ± 4	10.2
**N_2_**	1.8 ± 0.1	3.9 ± 0.7	3.5 ± 0.2	0.52 ± 0.06	8 ± 2	71
**O_2_**	2.8 ± 0.2	4.5 ± 0.7	3.1 ± 0.2	0.9 ± 0.1	5 ± 1	107
**CH_4_**	4.6 ± 0.3	7 ± 1	2.2 ± 0.2	2.1 ± 0.3	3 ± 1	149
**CO_2_**	17 ± 1	17 ± 2	2.4 ± 0.2	7.3 ± 0.9	2.3 ± 0.6	195
